# Behçet’s Syndrome and Thrombosis

**DOI:** 10.4084/MJHID.2011.026

**Published:** 2011-07-08

**Authors:** Emire Seyahi, Sebahattin Yurdakul

**Affiliations:** Department of Medicine, Division of Rheumatology, Cerrahpasa Medical Faculty, University of Istanbul, Istanbul, Turkey

## Abstract

Behçet syndrome (BS) is a multisystem vasculitis with unknown etiology and a unique geographic distribution. The disease course is characterized by exacerbations and remissions while abating as the years pass. The usual onset is in the third decade. Recurrent skin mucosa lesions and sight threatening panuveitis are the hallmark of the disease. Males are more severely affected than females. Vascular involvement can occur in up to 40% of cases. BS is unique among the vasculitides in that it may involve all sizes and types of vessels. It affects the veins more than the arteries. Lower extremity vein thrombosis is the most frequent manifestation of vascular involvement, followed by vena cava thrombosis, pulmonary artery aneurysms, Budd-Chiari syndrome, peripheral artery aneurysms, dural sinus thrombosis and abdominal aorta aneurysms. Vascular involvement is frequently associated with constitut onal symptoms and increased acute phase response and is the major cause of increased mortality. A predominantly neutrophilic vasculitis around the vaso vasorum is typical of BS. The thrombus is tightly adherent to the vessel wall which probably explains why thromboembolism is so rare despite the high frequency of venous disease. Thrombophilic factors do not seem to explain thrombotic tendency in BS. Immunosuppressive treatment is essential in suppression and preventing the attacks.

## Introduction:

We herein give first a general outline of Behçet’s syndrome (BS), then review the available data on the vascular part of the disease, and finally discuss the relative absence of embolic phenomena in spite of the high frequency of thrombotic episodes in this disorder in the Management section.

BS is named after Hulusi Behçet, a Turkish dermatologist, who described three patients with a triple symptom complex of aphthae, genital ulcers, and hypopyon uveitis for the first time in 1937.^1^ The syndrome is characterized by recurrent oral aphthae which is the sine qua non and other recurrent manifestations -in order of decreasing frequency-genital ulcers, variable skin lesions, arthritis, uveitis and thrombophlebitis.^2^,^3^ BS may also involve gastrointestinal and central nervous system.^2^,^3^ It is considered as a nonspecific systemic vasculitis of small and large vessels involving both venous and arterial sides. The aetiopathogenesis is still unknown.^2^–^5^

BS has a distinct geographical distribution along the ancient trading route known as the ‘*Silk Route*’, extending from the Mediterranean countries to Far East. This suggests that the etiological agent(s), including several genetic factors such as HLA-B51 had spread through this way.^6^ The prevalence of BS was reported to be between 20 to 421 among 100,000 *adult* population in Turkey while this was 17 in Iraq and 120 in an Arab community in Israel. The estimated prevalence ranges are less in other part of the globe: 0.64 in the UK, 6.4 in Spain, 7.1 in France, and 8.6 in the USA per 100,000.^7^ Some manifestations of the disease also show regional differences: gastrointestinal involvement is frequent in the Far East but infrequent in Turkey.^8^ The positivity of pathergy test is frequent in endemic countries whereas less common in Europe and the USA.^9^ Finally, the most consistent genetic marker, the HLA B51 association is more pronounced in the endemic areas as well.^7^

The usual onset of the syndrome is in the third decade. The onset is rare among the aged over 50 years and in the childhood. While both genders are equally affected the syndrome runs a more severe disease course among men and the young.^10^,^11^

A set of diagnostic (classification) criteria was published in 1990 by International Study Group.^12^ These criteria define oral ulceration as the sine qua non and additionally require two other organ involvements for the diagnosis, as shown in Table 1.

## Clinical Manifestations:

Clinical manifestations are variable and characterized by unpredictable periods of recurrences and remissions. Mucocutaneous features are the most common and the presenting symptoms of the disease whereas eye, vascular and neurological involvement are the most serious ones.

## Mucocutaneous Findings:

Oral ulcerations are frequently the first as well as the most frequent symptoms. Minor aphthous ulcers (<10mm in diameter) are the most common type. Aphthae are usually multiple and occur more frequently in BS but it is difficult to distinguish them from those of recurrent oral ulcers due to other causes.

Genital ulcers usually occur on the scrotum but are infrequent on the shaft or on the glans penis in males. Urethritis or dysuria is not a part of BS. Both major and minor labia are affected in the female. Genital ulcers affect the quality of life. The big ulcers usually heal with scarring, which is useful in differential diagnosis.^13^

Acne-like lesions or papulo-pustular lesions are seen both at the usual acne sites as well as at uncommon sites such as upper extremities and are also indistinguishable from acne vulgaris by both in appearance and pathologically.^14^ Nodular lesions are observed in 50 % of the patients and are usually confined to lower limbs. Erythema nodosum can be difficult to distinguish from superficial thrombophlebitis with the naked eye.

The pathergy reaction is a non-specific hyperreactivity of the skin to trauma such as a needle prick. A papule or pustule typically forms in 24–48 hours after a skin puncture with a needle. This is quite specific to Behçet’s patients.^15^ While the positivity in 60–70% of patients in Turkey and Japan it is rarely observed in patients with BS from Northern Europe and North America.^9^

Extra-genital ulcers, Sweet’s syndrome, pyoderma gangrenosum, leucocytoclastic vasculitis and true arterial lesions are other less common skin lesions.

## Eye Involvement:

A chronic, relapsing bilateral uveitis involving both anterior and posterior chambers are seen in half of all patients but is more frequent and more severe among the male and the young. Anterior uveitis with intense inflammation (hypopyon) observed in only a small fraction of patients indicates a bad outcome and is generally associated with severe retinal vasculitis. Posterior uveal inflammation with involvement of the retina can cause retinal exudates, haemorrhages, venous thrombosis, papilloedema and macular disease. Recurrent attacks of eye disease results in structural changes leading to loss of vision if left untreated.^16^

## Musculoskeletal System:

Joint involvement is observed in half of the patients. Arthritis is usually a non- deforming and non-erosive mono- or oligoarthritis resolving in a few weeks. The most frequently involved joints are knees, followed by ankles, wrist, and elbows.^17^ Back pain is rare and sacroiliac joint involvement is not part of the disease.^18^ Patients with BS and arthritis also have more acne lesions.^19^ Furthermore, patients with arthritis and acnea lesions have a significantly more entesopathy scores.^20^ Synovial fluid is commonly inflammatory but has a good mucin clot. Myositis can be seen rarely.

## Central Nervous System:

Central nervous system (CNS) disease occurs in 5–10 % of patients in the form of either parenchymal brain involvement (80 %) or in the form of non-parenchymal disease (20%) which is manifested as dural sinus thrombosis or intracranial hypertension. Brainstem involvement is the most characteristic type of involvement in the parenchymal type, while spinal cord and hemispheric involvement are rarely observed. Pyramidal signs, hemiparesis, behavioral –cognitive changes and sphincter disturbances and/or impotence are the main clinical manifestations. Psychiatric problems may develop in some patients. Peripheral neuropathy is rare. A high protein or cell count in cerebrospinal fluid examination implies a grave prognosis. On the other hand, non-parenchymal type of involvement - which will be discussed in detail in the vascular section - is presented mainly with symptoms of increased intracranial pressure symptoms such as severe headache, papilloedema and motor ocular nerve palsies.^21^,^22^ Dural sinus thrombosis has relatively benign prognosis.

## Gastrointestinal Involvement:

Gastrointestinal involvement occurs in one-third of patients from Japan^23^ but it is quite rare in Turkey.^8^ The symptoms resemble to those of inflammatory bowel diseases. Mucosal ulceration is found in the ileum, the caecum and the colon.^24^ Hepatic problems are not common in BS unless an associated Budd-Chiari syndrome is present.^25^

Other uncommon clinical manifestations are glomerulonephritis, amyloidosis of the AA type, voiding dysfunction due to direct bladder involvement, and epididymitis.

## Vascular Involvement:

Vascular disease is seen in up to 40 % of the patients and as seen in all major organ involvement it has a definite male preponderance.^11^,^26^ Venous involvement is more common than arterial disease (75 % vs 25 %).^11^,^26^ Lower extremity vein thrombosis (LEVT) is the most frequent manifestation of vascular involvement.^11^,^26^ Vena cava thrombosis, pulmonary artery aneurysms (PAA), Budd-Chiari syndrome, peripheral artery aneurysms, dural sinus thrombosis and abdominal aorta aneurysms were the other vascular manifestations as listed in order of decreasing frequency.^26^ Table 2 defines various forms of vascular involvement in BS. Arterial disease is manifested mostly in the form of aneurysms.^11^,^26^, ^27^ Arterial occlusions are seldom seen and reported to have a better prognosis than that of the arterial aneurysms.^27^

Vascular involvement is frequently associated with fever along with constitutional symptoms and manifest usually with high acute phase response. Furthermore, it causes severe morbidity and increased mortality.^11^ PAA, Budd-Chiari syndrome and vena cava thrombosis especially are the main diseases that are associated with increased mortality.

## Time to Occur:

LEVT is an early finding occurring usually within the first few years of disease onset.^11^,^26^ Similarly, PAA and Budd-Chiari syndrome are also reported to occur early.^11^,^26^,^28^ Dural sinus thrombosis also seems to occur early since it is the most preferred manifestation among juvenile BS patients.^21^,^22^,^29^ However, vena cava thrombosis and aneurysms other than PAA are late findings.^11^,^26^ We had reported that vena cava thrombosis develops in a median of 5 years whereas abdominal aorta and peripheral arterial aneurysms develop in a median of 7 years.^11^

## Types of involvement and clinical manifestations:

**Figure 1.**
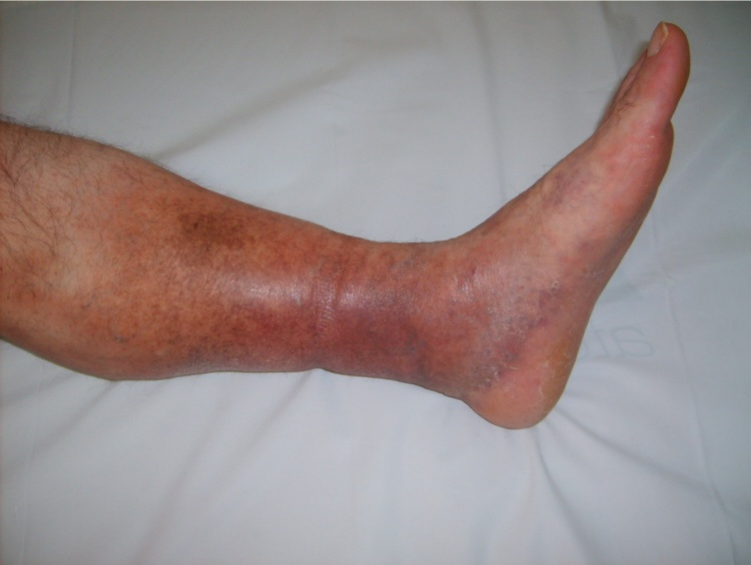
Chronic deep vein thrombosis on the lower extremity: Hyperpigmentation, edema, varicose veins and a mild induration with erythema are visible on the lower part of the tibia and foot.

**Figure 2.**
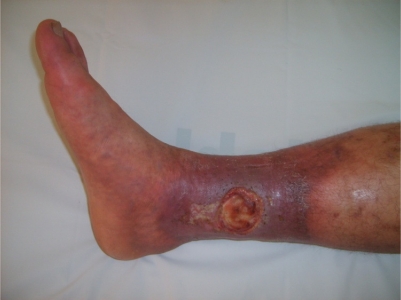
Chronic vein thrombosis with stasis ulcer on the lower extremity: Large active ulcer on the medial lower part of the tibia is noted in addition to the severe induration, hyperpigmentation and varicose veins on the skin.

a) *Lower extremity vein thrombosis (LEVT):* Deep veins of the lower extremity are the most common sites of venous thrombosis, which constitute 60–80 % of vascular lesions.^11^,^26^,^30^–^34^ The affected veins in descending order of frequency are femoral (superficial, deep and common), popliteal, saphenous (magna and parva) and crural veins.^35^ Furthermore, chronic relapsing vein thromboses in the legs tend to precede other sites of major vessel involvement.^26^ LVET may cause erythema with induration (77%), leg pain (74 %), varicose veins (68%), edema (61%), skin hyperpigmentation (58%), intermittent claudication (36%), and ulceration on the tibia or malleol (17%).^35^ Figure 1 and 2 show two BS patients with chronic vein thrombosis in the lower extremities. Recently, we assessed and compared clinical and radiologic characteristics of LVET between BS and non-BS patients.^35^ The mean number of involved veins and the clinical severity score were higher among BS patients with LVET compared to non-BS patients.^35^ Furthermore LVET tended to develop more bilaterally in BS patients compared to non-BS patients.^35^ It seems that venous thrombophlebitis and thrombotic involvement run a slow and insidious course over time, since many patients recall only one or two venous overt attacks in the legs, despite the radiological evidence of extensive progression of venous disease.^35^

Among the above mentioned clinical symptoms, intermittent claudication, we believe, deserves a bit detailed explanation. It is usually a sign of peripheral arterial insufficiency which is most of the time due to atherosclerosis. However, the ‘claudication’ symptom which we persistently find to be increased in BS patients with LEVT^35^,^36^ is stemming from chronic thrombosis of iliac/femoral veins and must be described in fact as “venous claudication” which is defined by several authors in the past as exercise related thigh or leg pain resulting from severe venous outflow impairment.^37^ Moreover we and others showed that atherosclerosis was not increased in BS.^36^,^38^–^40^

*b) Superficial thrombophlebitis:* Superficial thrombophlebitis (STM) tends to be associated with deep vein thrombosis in the lower extremity and inferior vena cava.^41^,^42^ It is manifested as red nodular lesions that mimic erythema nodosum.^3^ While clinically the two lesions are indistinguishable from each other erythema nodosum is more common in females and associated usually with other mucocutaneous manifestations such as oral and genital ulcer.^42^ On the other hand STM lesions are more common among male patients and associated with large vessel involvement elsewhere.^41^,^42^ On closer examination STM lesions are seen as string –like lesions following vein tracts. Both B-mode and Doppler dermal ultrasonography can be helpful to differentiate between the two.^42^

STM involve large and small veins of the lower extremities, major saphenous vein being mostly affected. Histological examination reveals organized thrombi in the lumen of the involved vein. On the other hand septal pannuculitis with medium vessel vasculitis is frequently seen in the histopathology specimens of erythema nodosum.^43^

**Figure 3.**
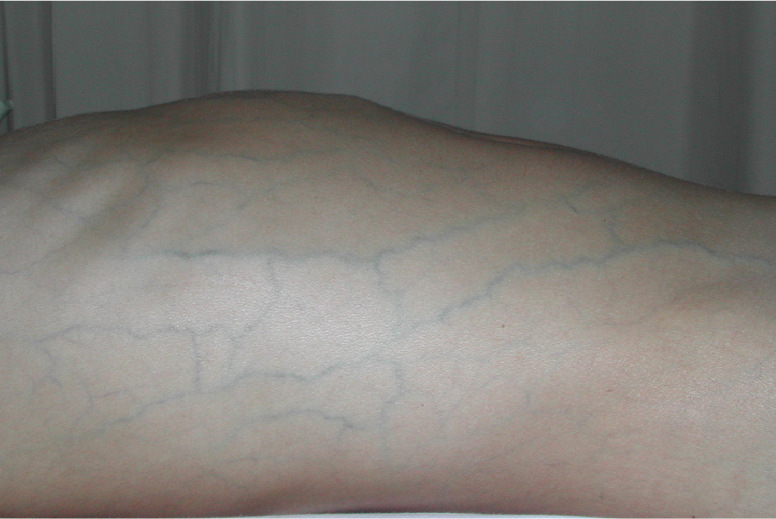
Collaterals on the abdominal wall in a patient with vena cava inferior thrombosis and Budd-Chiari syndrome. Note the profuse swelling and distention of the abdomen due to ascites.

c) *Vena cava thrombosis:* Clinical signs vary according to the anatomical localization of the involvement. Chronic occlusion of the caval systems leads to the appearance of prominent venous collaterals on the thoracic and abdominal walls (Figure 3). Obstruction of inferior vena cava (IVC) may cause venous claudication, crural ulcers, oesophageal varices, and hyperpigmentation on the skin of the lower extremities.^26^,^30^–^34^ Thrombotic involvement may extend from hepatic veins to femoral / iliac veins.^35^

**Figure 4.**
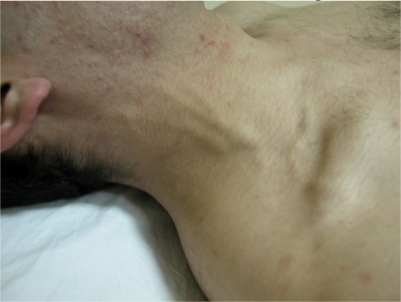
Neck collaterals in a patient with vena cava superior thrombosis.

Superior vena cava (SVC) thrombosis presents with swelling in the face and upper extremities with full jugular veins without pulsation (Figure 4).^26^,^30^–^34^ Occasionally patients may have dyspnea and sleep apnea disorder. Lower extremity deep vein thrombosis is less common than in patients with IVC disease.^26^ Despite the alarming presentation, the SVC thrombosis in BS usually has a benign course with efficient collateral circulation.^44^ It might rarely be complicated with pleural effusion and chylothorax.^44^

d) *Hepatic veins:* Hepatic vein thrombosis may cause Budd-Chiari syndrome which may manifest clinically as abdominal pain, ascites, and edema on the scrotum and lower extremities (Figure 3). Liver failure may develop in severe cases. It is a rare complication of BS, but carries a high mortality rate. In one large series from Turkey surveyed by Bayraktar et al, the frequency and outcome of Budd-Chiari syndrome in 493 patients with BS during a 8 year period from 1985 to 1994 was studied.^28^ BS was the single most frequent cause of Budd Chiari syndrome, accounting for roughly half of such patients.^28^ There were 14 (26%) patients with Budd-Chiari syndrome out of 53 patients with large vessel thrombosis. Of these 14 patients 10 (60%) died with a mean survival of 10 months.^28^ In our 20 year survey all 3 patients with Budd-Chiari syndrome had died during the follow-up.^11^

e) *Dural sinus thrombosis:* Thrombosis of the venous sinuses may present with symptoms of increased intracranial pressure such as severe headache, papilloedema, sixth nerve palsy and rarely with fever. The major vessel involvement is closely associated with dural sinus thrombi, suggesting that this type of neurological involvement is also part of the vascular spectrum.^45^ Dural sinus thrombosis is also the predominant type of neurological involvement in juvenile BS patients.^29^ This type of neurological involvement has a significantly favorable outcome than parenchymal type and is seen mostly in males.^21^,^22^

f) *Pulmonary artery involvement:* Pulmonary artery involvement (PAI) is uncommon with a reported prevalence rate of less than 5%.^46^,^47^ PAI is mainly manifested by pulmonary artery aneurysms (PAA) and less often solely by ‘in situ’ pulmonary artery thrombosis (PAT) in CT images.^48^ Thrombosis develops usually as a complication to underlying extensive vasculitis. Therefore we suppose that despite the high prevalence of venous thrombosis in BS as presented earlier, pulmonary thromboembolism is extremely rare in BS. Our clinical studies support this assumption by not finding any pulmonary thromboembolism case among BS with extensive venous disease when followed longitudinally for a substantial period of time.^11^,^26^,^35^,^46^–^48^ Also, one Japanese study investigated autopsies of the 170 patients with BS (122 M/ 48 F) and found no single case with pulmonary thromboembolism.^49^ On the other hand, ‘in situ’ PAT in BS has a similar clinical and prognostic picture compared to PAA.^48^

Patients with PAI present with fever, chest pain, coughing, dyspnea and hemoptysis.^48^ It is always associated with high acute phase response.^48^ PAA are observed as bilateral or unilateral hilar opacities on chest X-ray or thorax CT scans. Aneurysms can be partially or totally thrombosed in about third of the cases.^48^ The involvement is usually bilateral and confined to main, lobar or segment arteries.^47^–^48^ Inferior lobes are mostly involved.^48^

PAI causes significant morbidity and mortality.^11^,^46^,^47^ We had reported in 1994 that 12 patients with PAA out of 24 (all men) died a mean of 10 months after the onset of haemoptysis.^46^ A decade later in 2004, we updated the outcome of PAA with 26 BS patients who had been followed between 1992 and 2002.^47^ There were significantly less deaths (23%) during a mean of 4 years in the recent group attributed mainly to earlier recognition and prompt treatment.^47^

g) *Peripheral arteries:* Aortic and peripheral arterial aneurysms are also major causes of death because of the risk of rupture. Twenty-four patients (all male) with either abdominal aorta or peripheral artery aneurysms were identified between 1977 and 1996 at the thoracic and cardiovascular surgery department of Cerrahpasa Medical Faculty in a study by Tuzun et al.^50^ Mortality was reported to be 17%.^50^ Mostly abdominal aorta, femoral, iliac, popliteal and carotid arteries are involved.^50^ Clinical signs of abdominal aortic aneurysms include abdominal or back pain. Peripheral aneurysms present with pulsatile masses in the extremities or the neck. Constitutional symptoms like low grade fever, loss of appetite or an increase in the acute phase response are additional signs.^50^

h) *Intracardiac thrombosis:* Intracardiac thrombosis is a rare finding in BS, reported mostly in case reports.^51^ It is strongly associated with vascular involvement elsewhere in the body, mostly with PAI.^51^ It is found more frequently among young males and located usually in the right side of the heart, right ventricle being the most common place.^51^ The thrombosis was reported to be tightly adhered to underlying endocardium or myocardium.^51^ Histological studies revealed that there was an organizing thrombus containing inflammatory cell infiltrates with or without involvement of underlying cardiac tissue.^51^

## Histopathology of Vascular Disease:

Vascular inflammation is diffuse not patchy, involving large segments of the vessel wall.^52^ This is usually associated with superimposed thrombus.^52^,^53^ Thrombus as a rule is tightly adherent to the vessel wall supposedly without a free floating tail.^52^ The veins in dermis and subcutis may be obliterated with organizing thrombi.^53^ A predominantly neutrophilic vasculitis around the vaso vasorum is typical of BS.^50^,^52^,^53^ Other than that, vascular walls may show fibrous thickening, accompanied by non-specific inflammatory infiltrate.^53^ Kobayashi et al reported that there were intact internal elastic membrane and increased expression of HLA-DR positive cells in the endothelium.^54^ Tuzun et al however, reported that in addition to the inflammation around the vaso vasorum, there were severe medial destruction with loss of elastic and muscle fibers, disarray of the internal elastic membrane in the active stage and dense periadventitial fibrosis in the chronic stage.^50^ He also observed reactive lymph nodes that are found in the immediate vicinity of the peripheral arterial aneurysms.^50^

## Coagulation Abnormality in BS:

So far, none of the thrombophilic factors were shown to be associated with the thrombotic tendency observed in BS.^52^,^55^–^59^ A defect in fibrinolysis was however suggested.^60^,^61^ Gul et al previously reported that coagulation factor V gene G1691A mutation (factor V Leiden), could contribute to thrombotic complications in BS patients with deep vein thrombosis.^62^ However, this was not confirmed.^56^,^63^,^64^ The current data indicates that the pathogenesis of thrombosis in BS is not due to a coagulation abnormality.

## Management of Vascular Disease:

The role of anticoagulation in deep vein thrombosis has not been evaluated in a controlled study. However, 2 retrospective studies showed that anti-coagulant treatment is ineffective in preventing venous thrombosis.^65^,^66^ One study from Turkey, investigated the long term course of deep vein thrombosis in 95 BS patients.^65^ The recurrence was observed in 44 (46%) and post-thrombotic syndrome in 21 (22%) of the patients. The risk for recurrent DVT and development of the post-thrombotic syndrome was found to be significantly reduced in patients receiving immunosuppressant drugs (azathioprine or cyclophosphamide) (OR= 0.3, 95% CI: 0.04–0.88, p = 0.03, and OR = 0.2, 95% CI= 0.04–0.88, p = 0.03, respectively), whereas, anticoagulants were not found to be effective (OR= 0.6, 95% CI: 0.19–1.9, p = 0.4).^65^ One retrospective Korean study compared anticoagulant and immunosuppressive treatment in 37 BS patients with venous thrombosis (66). BS patients with venous thrombosis were divided into three groups: one group (n = 16) received immunosuppressive therapy alone, another group (n = 17) received immunosuppressant and anticoagulation combination therapy, and the third group (n = 4) received anticoagulation therapy only. Recurrence of venous thrombosis occurred in two cases in the immunosuppressant group (12.5%), one case in the combination therapy group (5.9%), and three cases in the anticoagulant group (75%). No significant difference was found between recurrence in the immunosuppressant and combination therapy groups. The study suggested that immunosuppressive therapy is essential and that anticoagulation therapy might not be required for the treatment of deep venous thrombosis associated with BS.^66^

Because of inefficacy of the anticoagulation shown in these two retrospective studies^65^,^66^ and due to the reasons that we discussed earlier [a. relative absence of embolic phenomena in spite of the thrombotic episodes in our clinical experience with over 7000 patients during 30 years and in the large Japanese autopsy registry, b. sticky nature of thrombosis with no floating free tail, c. absence of coagulation abnormality, d. strong association of life threatening PAA with deep vein thrombosis and e. the effectiveness of azathioprine in preventing thrombotic attacks based on the results observed in both azathioprine trials]^67^,^68^ we prefer not to anti-coagulate BS patients with venous thrombosis. Our general approach is to treat these patients with immunosuppressive agents.^69^ Furthermore, experience with fibrinolytic therapy is scarce and has also been unsuccessful. Surgery of venous thrombosis is not advocated.

Arterial aneurysms, especially pulmonary arterial aneurysms carry a more severe prognosis than venous thrombosis. They can rupture or fistulate into the bronchi causing massive hemoptysis and even death in about 23% of the cases.^46^–^48^ We suggest monthly pulses of cyclophosphamide combined with 1mg/kg of prednisolone and tapering the prednisolone dose to <10 mg/day after three months. Anticoagulation is contraindicated because of the risk of bleeding. Our experience with intra-arterial embolisation is limited, however should be tried in treatment resistant cases. Surgical resection is also not successful as often PAA are multiple and located at different parts of the lungs. Aneurysms of the peripheral arteries should be corrected surgically although there is a recurrence rate of about 30%.^50^ Ligations were defined as the preferred choice of surgical treatment for aneurysms localized in the extremities while, abdominal aortic aneurysms were treated better with graft insertions.^50^ It has been also suggested that immunosuppressive treatment should be given to prevent recurrences.

## Figures and Tables

**Table 1.** International Study Group Criteria for the Diagnosis of Behçet’s Syndrome

**Criterion**	**Definition**
Recurrent oral ulceration	Aphthous or herpetiform lesions; observed by the physician or patient; recurring at least 3 times a year.
Recurrent genital ulceration	Aphthous ulceration or scarring observed by the physician or reliably described by the patient.
Eye lesions	Anterior or posterior uveitis or cells in the vitreus body on slit –lamp examination or retinal vasculitis detected by an ophthalmologist.
Skin lesions	Erythema nodosum, pseudofolliculitis, papulopustular lesions or acneiform nodules, not related to glucocorticoids treatment or adolescence.
Positive pathergy test	Test interpreted as positive by the physician at 24–48 h.

**Table 2.** Various forms of vascular involvement in Behçet’s syndrome

Deep vein thrombosis of lower extremities Femoral with or without iliac vein Popliteal vein Crural vein Superficial thrombophlebitis Greater saphenous vein Lesser saphenous vein Superior vena cava syndrome Inferior vena cava syndrome Cerebral venous sinus thrombosis Budd-Chiari syndrome Abdominal aortic aneurysm Peripheral aneurysm Femoral/Iliac artery Popliteal artery Tibial artery Radial artery Carotid artery aneurysm Pulmonary artery aneurysm/thrombosis Other rare venous & arterial events Brachial vein thrombosis Portal vein thrombosis Radial artery occlusion

## References

[1] Behcet H (1937). Uber rezidivierende, aphthose, dürch ein Virus verursachte Geshwure am Munde, am Auge und an den Genitalien. Dematologische Wochenschrift.

[2] Sakane T, Takeno M, Suzuki N, Inaba G (1999). Behcet’s disease. New Eng J Med.

[3] Yurdakul S, Hamuryudan V, Fresko I, Yazıcı H, Hochberg MC, Silman AJ, Smolen YS, Weinblatt ME, Weisman MH (2011). Behçet’s syndrome. Rheumatology.

[4] Direskeneli H, Saruhan-Direskeneli S, Yazici Y, Yazici H (2010). Disease Mechanisms. Behçet’s Syndrome.

[5] Gul A, Ohno S, Yazici Y, Yazici H (2010). Genetics of Behçet’s disease. Behçet’s Syndrome.

[6] Verity DH, Marr JE, Ohno S (1999). Behçet’s disease, the Silk Road and HLA-B51: historical and geographical perspectives. Tissue Antigens.

[7] Yurdakul S, Yazici Y, Yazici Y, Yazici H (2010). Epidemiology of Behçet’s syndrome and regional differences in disease expression. Behçet’s Syndrome.

[8] Yurdakul S, Tuzuner N, Yurdakul I (1996). Gastrointestinal involvement in Behçet’s syndrome: a controlled study. Ann Rheum Dis.

[9] Yazıcı H, Chamberlain MA, Tuzun Y (1984). A comparative study of the pathergy among Turkish and British patients with Behçet’s disease. Ann Rheum Dis.

[10] Yazici H, Tuzun Y, Pazarli H (1984). Influence of age of onset and patient’s sex on the prevalence and severity of manifestations of Behçet’s syndrome. Ann Rheum Dis.

[11] Kural-Seyahi E, Fresko I, Seyahi N (2003). The long-term mortality and morbidity of Behcet syndrome: a 2-decade outcome survey of 387 patients followed at a dedicated center. Medicine (Baltimore).

[12] International Study Group for Behçet’s Disease (1990). Criteria for diagnosis of Behçet’s disease. Lancet.

[13] Mat C, Göksugur N, Engin B (2006). The frequency of scarring after genital ulcers in Behçet’s syndrome: a prospective study. Int J Dermatol.

[14] Ergun T, Gurbuz O, Dogusoy G (1998). Histopathologic features of the spontaneous pustular lesions of Behçet’s syndrome. Int J Dermatol.

[15] Tüzün Y, Yazıcı H, Pazarli H (1979). The usefulness of the nonspecific skin hyperreactivity (The pathergy test) in Behçet’s disease in Turkey. Acta Dermatovener (Stockholm).

[16] Tugal-Tutkun I, Onal S, Altan-Yaycioglu R (2004). Uveitis in Behçet disease: an analysis of 880 patients. Am J Ophthalmol.

[17] Yurdakul S, Yazici H, Tuzun Y (1983). The arthritis of Behçet’s disease: a prospective study. Ann Rheum Dis.

[18] Yazici H, Tuzlaci M, Yurdakul S (1981). A controlled survey of sacroiliitis in Behçet’s disease. Ann Rheum Dis.

[19] Diri E, Mat C, Hamuryudan V (2001). Papulopustular skin lesions are seen more frequently in patients with Behçet’s syndrome who have arthritis: a controlled and masked study. Ann Rheum Dis.

[20] Hatemi G, Fresko I, Tascilar K, Yazici H (2008). Enthesopathy is increased among Behçet’s syndrome patients with acne and arthritis: an ultrasonographic study. Arthritis Rheum.

[21] Akman-Demir G, Serdaroglu P, Tasci B (1999). Clinical patterns of neurological involvement in Behçet’s disease: evaluation of 200 patients. The Neuro-Behcet Study Group. Brain.

[22] Siva A, Kantarci OH, Saip S (2001). Behçet’s disease: diagnostic and prognostic aspects of neurological involvement. J Neurology.

[23] Shimizu T, Ehrlich GE, Inaba G, Hayashi K (1979). Behçet disease (Behçet syndrome). Semin Arthritis Rheum.

[24] Korman U, Cantasdemir M, Kurugoglu S (2003). Enteroclysis findings of intestinal Behcet disease: a comparative study with Crohn disease. Abdom Imaging.

[25] Bayraktar Y, Ozaslan E, Van Thiel DH (2000). Gastrointestinal manifestations of Behcet’s disease. J Clin Gastroenterol.

[26] Melikoglu M, Ugurlu S, Tascilar K (2008). Large Vessel Involvement in Behcet’s Syndrome: A Retrospective Survey. Ann Rheum Dis.

[27] Hamza M (1987). Large artery involvement in Behcet’s disease. J Rheumatol.

[28] Bayraktar Y, Balkanci F, Bayraktar M (1997). Budd-Chiari syndrome: a common complication of Behçet’s disease. Am J Gastroenterol.

[29] Seyahi E, Ozdogan H, Uğurlu S (2004). The outcome children with Behçet’s syndrome. Clin Exp Rheumatol.

[30] Koc Y, Gullu I, Akpek G (1992). Vascular involvement in Behcet’s disease. J Rheumatol.

[31] Tohmé A, Aoun N, El-Rassi B (2003). Vascular manifestations of Behçet’s disease. Eighteen cases among 140 patients. Joint Bone Spine.

[32] Sarica-Kucukoglu R, Akdag-Kose A, Kayabali M (2006). Vascular involvement in Behçet’s disease: a retrospective analysis of 2319 cases. Int J Dermatol.

[33] Düzgün N, Ateş A, Aydintuğ OT (2006). Characteristics of vascular involvement in Behçet’s disease. Scand J Rheumatol.

[34] Chae EJ, Do KH, Seo JB (2008t). Radiologic and clinical findings of Behçet disease: comprehensive review of multisystemic involvement. Radiographics.

[35] Cakmak OS, Seyahi E, Kantarci F (2010). Venous severity assessment in Behçet’s syndrome. Clin Exp Rheumatol.

[36] Ugurlu S, Seyahi E, Yazici H (2008). Prevalence of angina, myocardial infarction and intermittent claudication assessed by Rose Questionnaire among patients with Behcet’s syndrome. Rheumatology.

[37] Walker RT, Woodyer AB, Dormandy JA (1985). Venous claudication. A report of 15 cases and a review of the literature. Int Angiol.

[38] Seyahi E, Memisoglu E, Hamuryudan V (2004). Coronary atherosclerosis in Behcet’s syndrome: a pilot study using electron-beam computed tomography. Rheumatology (Oxford).

[39] Seyahi E, Ugurlu S, Cumali R (2008). Atherosclerosis in Behçet’s Syndrome. Semin Arthritis Rheum.

[40] Rhee MY, Chang HK, Kim SK (2007). Intima-media thickness and arterial stiffness of carotid artery in Korean patients with Behçet’s disease. J Korean Med Sci.

[41] Tunc R, Keyman E, Melikoglu M, Fresko I, Yazici H (2002). Target organ associations in Turkish patients with Behçet’s disease: a cross sectional study by exploratory factor analysis. J Rheumatol.

[42] Yazici H (2004). The lumps and bumps of Behcet’s syndrome. Autoimmun Rev.

[43] Demirkesen C, Tuzuner N, Mat C (2001). Clinicopathologic evaluation of nodular cutaneous lesions of Behcet syndrome. Am J Clin Pathol.

[44] Hamuryudan V, Melikoglu M, Yazici Y, Yazici H (2010). Vascular involvement in Behçet’s syndrome. Behçet’s Syndrome.

[45] Tunc R, Saip S, Siva A, Yazici H (2004). Cerebral venous thrombosis is associated with major vessel disease in BS’s syndrome. Ann Rheum Dis.

[46] Hamuryudan V, Yurdakul S, Moral F (1994). Pulmonary arterial aneurysms in Behçet’s syndrome: a report of 24 cases. Br J Rheumatol.

[47] Hamuryudan V, Er T, Seyahi E (2004). Pulmonary artery aneurysms in Behcet syndrome. Am J Med.

[48] Seyahi E, Melikoglu M, Akman C (2007). Pulmonary vascular involvement in Behcet’s syndrome. Arthritis Rheum.

[49] Lakhanpal S, Tani K, Lie JT (1985). Pathologic features of Behçet’s syndrome: a review of Japanese autopsy registry data. Hum Pathol.

[50] Tüzün H, Beşirli K, Sayin A, Yazici H (1997). Management of aneurysms in Behçet’s syndrome: an analysis of 24 patients. Surgery.

[51] Mogulkoc N, Burgess MI, Bishop PW (2000). Intracardiac thrombus in Behçet’s disease: a systematic review. Chest.

[52] Fresko I, Melikoglu M, Tunc R, Gene V, Ball S, Bridges Louis (2002). Behcet’s syndrome: pathogenesis, clinical manifestations and treatment in Vasculitis.

[53] Demirkesen C, Oz B, Goksel S, Yazici Y, Yazici H (2010). Histopathology of Behcet’s syndrome. Behçet’s Syndrome.

[54] Kobayashi M, Ito M, Nakagawa A (2000). Neutrophil and endothelial cell activation in the vasa vasorum in vasculo-Behçet disease. Histopathology.

[55] Sengül N, Demirer S, Yerdel MA (2000). Comparison of coagulation parameters for healthy subjects and Behçet disease patients with and without vascular involvement. World J Surg.

[56] Espinosa G, Font J, Tassies D (2002). Vascular involvement in Behcet’s disease: relation with thrombophilic factors, coagulation activation, and thrombomodulin. American Journal of Medicine.

[57] Leiba M, Seligsohn U, Sidi Y (2004). Thrombophilic factors are not the leading cause of thrombosis in Behcet’s disease. Annals of the Rheumatic Diseases.

[58] Lee YJ, Kang SW, Yang JI (2002). Coagulation parameters and plasma total homocysteine levels in Behcet’s disease. Thromb Res.

[59] Mader R, Ziv M, Adawi M (1999). Thrombophilic factors and their relation to thromboembolic and other clinical manifestations in Behçet’s disease. J Rheumatol.

[60] Yurdakul S, Hekim N, Soysal T (2005). Fibrinolytic activity and d-dimer levels in Behçet’s syndrome. Clin Exp Rheumatol.

[61] Ricart JM, Ramón LA, Vayá A (2008). Fibrinolytic inhibitor levels and polymorphisms in Behçet disease and their association with thrombosis. Br J Haematol.

[62] Gül A, Özbek U, Öztürk C, Inanç M, Koniçe M, Ozçelik T (1996). Coagulation factor V gene mutation increases the risk of venous thrombosis in Behçet’s disease. Br J Rheumatol.

[63] Toydemir PB, Elhan AH, Tükün A (2000). Effects of factor V gene G1691A, methylenetetrahydrofolate reductase gene C677T, and prothombin gene G20210A mutations on deep venous thrombogenesis in Behçet’s disease. J Rheumatol.

[64] Silingardi M, Salvarani C, Boiardi L (2004). Factor V Leiden and prothrombin gene G20210A mutations in Italian patients with Behçet’s disease and deep vein thrombosis. Arthritis Rheum.

[65] Kahraman O, Celebi-Onder S, Kamali S (2003). Long-term course of deep venous thrombosis in Patients with Behçet’s disease. Arthritis Rheum.

[66] Ahn JK, Lee YS, Jeon CH (2008). Treatment of venous thrombosis associated with Behcet’s disease: immunosuppressive therapy alone versus immunosuppressive therapy plus anticoagulation. Clin Rheumatol.

[67] Yazici H, Pazarli H, Barnes CG (1990). A controlled trial of azathioprine in Behcet’s syndrome. N Engl J Med.

[68] Hamuryudan V, Ozyazgan Y, Hizli N (1997). Azathioprine in Behcet’s syndrome: effects on long-term prognosis. Arthritis Rheum.

[69] Hatemi G, Silman A, Bang D (2009). Management of Behçet disease: a systematic literature review for the European League Against Rheumatism evidence-based recommendations for the management of Behçet disease. Ann Rheum Dis.

